# Validation of the Malay Version of the Inventory of Functional Status after Childbirth Questionnaire

**DOI:** 10.1155/2015/972728

**Published:** 2015-01-15

**Authors:** Norhayati Mohd Noor, Aniza Abd. Aziz, Mohd Rosmizaki Mostapa, Zainudin Awang

**Affiliations:** ^1^Department of Family Medicine, School of Medical Sciences, Universiti Sains Malaysia, Health Campus, 16150 Kubang Kerian, Kelantan, Malaysia; ^2^Fakulti Perubatan dan Sains Kesihatan, Universiti Sultan Zainal Abidin, Kampus Kota, Jalan Sultan Mahmud, 20400 Kuala Terengganu, Terengganu, Malaysia; ^3^Universiti Institut Teknologi Mara, 15200 Kota Bharu, Kelantan, Malaysia

## Abstract

*Objective*. This study was designed to examine the psychometric properties of Malay version of the Inventory of Functional Status after Childbirth (IFSAC). *Design*. A cross-sectional study. *Materials and Methods*. A total of 108 postpartum mothers attending Obstetrics and Gynaecology Clinic, in a tertiary teaching hospital in Malaysia, were involved. Construct validity and internal consistency were performed after the translation, content validity, and face validity process. The data were analyzed using Analysis of Moment Structure version 18 and Statistical Packages for the Social Sciences version 20. *Results*. The final model consists of four constructs, namely, infant care, personal care, household activities, and social and community activities, with 18 items demonstrating acceptable factor loadings, domain to domain correlation, and best fit (Chi-squared/degree of freedom = 1.678; Tucker-Lewis index = 0.923; comparative fit index = 0.936; and root mean square error of approximation = 0.080). Composite reliability and average variance extracted of the domains ranged from 0.659 to 0.921 and from 0.499 to 0.628, respectively. *Conclusion*. The study suggested that the four-factor model with 18 items of the Malay version of IFSAC was acceptable to be used to measure functional status after childbirth because it is valid, reliable, and simple.

## 1. Background

Women assume many roles in their lifetime with motherhood being among the most difficult and time-consuming. Functional status after childbirth, defined as mother's readiness to assume infant care responsibilities as well as resuming personal care, household, social, community, and occupational activities [1] was measured using Inventory of Functional Status after Childbirth (IFSAC). IFSAC is one of the best tools to measure functional status after childbirth as it took into account the unique situation of recovery from childbirth and, thus, is useful for health personnel especially midwives.

The original conceptual model of IFSAC was the Roy's adaptation model that was developed by Sister Callista Roy. The overall goal is to focus on promoting health of the individual and group by promoting adaptation in physiological-physical, self-concept, role function, and interdependence modes [2]. The IFSAC was designed to measure functional status in the specific situation of recovery from childbirth [1]. The five-dimensional subscales contained within the instrument are infant care; personal care; household activities; social and community activities; and occupational activities. It does not measure feelings about such roles, only whether activities associated with the role have or have not begun or resumed. An assumption fundamental to IFSAC is that mothers will keep most, if not all, of their old roles; however, the level of performance in these roles may change. Evaluation of IFSAC showed content validity to be 84.4%, interrater reliability 97%, and Cronbach's alpha of 0.79 for total IFSAC with subscale coefficients for infant care of 0.92; personal care of 0.56; household of 0.64; social and community of 0.67; and occupational of 0.98 in a sample of 76 women tested [1]. It was validated in Australia with Cronbach's alpha of 0.81 [3] and 0.75 in Turkey [4].

McVeigh and Chaboyer  (2002) have generated correlational matrices in the analysis of 173 women to examine the construct validity of the IFSAC subscales using an item total correlation of 0.3 reporting that items in household and infant care were correlated. While the correlations of item of personal care were very weak for many of the combinations and only two of the 28 bivariate correlations were stronger, occupational had one item that had very weak correlations with other items [5]. Validity was supported for household and infant care and social and community at 6 months [5].

Confirmatory factor analysis (CFA) is a theory-testing model as opposed to a theory-generating method like exploratory factor analysis [6]. It begins with a hypothesis prior to the analysis which is based on a strong theoretical and/or empirical foundation [7]. According to Brown (2006), CFA is a type of structural equation modelling (SEM) that specifically deals with measurement models, that is, the relationships between observed measures or indicators and latents variables or factors [8]. It is powerful because it provides explicit hypothesis testing for factor analytic problems.

Assessment of functional status post-delivery is especially important for continuity of care. Despite this, the reliability and validity of IFSAC in the Malaysian culture have not been established. Here, the study aimed to determine the psychometric properties of the Malay version of IFSAC among postpartum mothers in a tertiary teaching hospital in Malaysia.

## 2. Methodology

### 2.1. Translational Process

The forward and backward translation was carried out by a group of experts whom consisted of researchers, linguists, and physicians. The process of translation has been carefully planned with the importance of ensuring the preservations of the meanings and followed by the content validity and face validity processes. Content validity refers to adequacy of the content of an instrument in terms of the number and scope of the individual questions that it contains; face validity refers to checking whether items in an instrument appear “on the face of it” to cover the intended topics clearly and unambiguously [9].

### 2.2. Data Collection

A cross-sectional study was conducted in Kelantan from September 2012 to April 2013 among postpartum women aged 18 years and older. Illiterates were excluded. Convenient sampling was applied. Sample size for CFA depends on the model complexity and basic measurement model characteristics. Hair et al. (2010) have suggested a minimum sample of 100 for a model with five or less latent constructs and more than three items in each latent construct [10]. The personal administered Malay version of IFSAC questionnaire was distributed to mothers at 1-month postpartum [3, 11, 12] attending Obstetrics and Gynaecology clinic, Universiti Sains Malaysia. The study was approved by Human Research Ethic Committee of Universiti Sains Malaysia and written consent was obtained from all the participants.

### 2.3. Research Tool

The IFSAC questionnaire was developed by Fawcett et al.   (1988). The IFSAC consists of five subscales and 36 items, that is, infant care (6 items), personal care (8 items), household activities (12 items), social and community activities (6 items), and occupational activities (4 items). The questionnaires are rated on a 4-point Likert scale and mean scores were calculated with one point being the lowest and four points being the highest scores possible. The higher the mean score, the greater the functional status.

For household's care domain's item response, one point was given to “never did as before childbirth,” and two points were given for “beginnings do as before childbirth,” three points for “do mostly as before childbirth,” and four points for “do every time as before childbirth.” For social activities domain's item response, one point was given to “never did as before childbirth,” and two points were given for “beginnings do as before childbirth,” three points for “do mostly as before childbirth,” four points for “do every time as before childbirth.” For baby's care domain's item response, one point was given to “never,” and two points were given for “sometime,” three points for “mostly,” and four points for “every time.” For personal care domain's item response, one point was given to “never,” and two points were given for “sometime,” three points for “mostly,” and four points for “every time” but reverse for items 25, 26, 27, 28, and 32. For occupational activities domain's item response, one point was given to “never,” and two points were given for “sometime,” three points for “mostly,” and four points for “every time” but reverse for items 34 and 35.

### 2.4. Statistical Analysis

Data entry and internal consistency reliability analysis were conducted using Statistical Packages for the Social Sciences (SPSS) version 20 and CFA for validity assessment was conducted using Analysis of Moment Structure (AMOS) version 18. Factor analysis was used for data reduction for overall scale and subscales of infant care, personal care, household activities, social, community activities, and occupational activities.

CFA with five latent constructs was specified for the analysis. Convergent validity is achieved when all items in a measurement model are statistically significant and average variance extracted (AVE) of ≥0.5. AVE is the average percentage of variation explained by the items in a construct. For construct validity, several goodness-of-fit indicators, including goodness of fit index (GFI), comparative fit index (CFI), Tucker-Lewis index (TLI) of >0.9, *χ* ^2^/df of <5.0, indicate acceptable level. The root mean square error of approximation (RMSEA) tests the fit of the model to the covariance matrix and value of <0.08 is an acceptable fit [13]. In addition to the overall evaluation of goodness of fit, the standardized factor loadings were examined in order to identify the misspecification for model modification. Factor loading of more than 0.6 is acceptable. Discriminant validity is achieved when the measurement model is free from redundant items [14].

Reliability was estimated by the internal consistency and Cronbach's alpha coefficient of ≥0.70 was considered satisfactory [15]. Construct reliability (CR) is an internal consistency of the measured variables representing a latent construct. Values of CR ≥ 0.6 and AVE ≥ 0.5 are required [14].

Concurrent criterion validity was performed against the physical component summary (PCS) of 12-item Short Form Health survey (SF-12). SF-12 is a shorter version of SF-36, that is, a well-known generic health-related quality of life measure used worldwide [16]. Cross-cultural validation studies have shown that there were substantial correlations between the summary measures of the SF-36 and the SF-12 Health survey [17]. The Malay version of SF-12v2 Health survey was prepared by the International Quality of Life Assessment (IQOLA) Project using the standard IQOLA translation methodology [18] with the Cronbach's alpha of 0.749 [19]. The Malay version of SF-12v2 Health survey was distributed simultaneously to the participants along with the translated IFSAC questionnaire. Pearson's product moment correlation coefficient (*r*) was applied to establish the criterion validity.

## 3. Results

A total of 108 postpartum mothers responded. The sociodemographic characteristics of the respondents were as shown in Table 2. All of the respondents were Malays with mean (SD) age of 31.4 (6.02) years.

### 3.1. Content and Face Validity

The experts assessed content validity after the translation process. The experts consist of a family health specialist, an obstetrics and gynaecology specialist, and a public health physician. Respondent testing was done among ten female staffs that experienced delivery in the study facility to fulfill the face validity process. They were required to review and comment the whole questionnaire in terms of its presentation, arrangement, clarity, and relatedness. Their understandings on each of item were also explored. They were satisfied with the presentation and clarity but had difficulty to understand all items in the social activities domains. Therefore, local examples were added to each item in order to enhance the meaning of the items. A similar approach was taken for three items in the household's care domain in which the Malay women respondents perceived them as having similar meaning. The examples were item B1.2: cleaning the house (e.g., sweeping and mopping the floor), item B1.3: tidying the house (e.g., tidy up bed and arrange scattered house items), and item B1.11: heavy housework and maintenance work (e.g., painting and washing curtains) (Table 1).

### 3.2. Normality Assessment

Univariate normality checking showed histogram and Q-Q plot of 36 items were normally distributed. However, the box-and-whisker plots showed that there were six items with extreme outlier, namely, item B3.19 with four extreme outliers, B3.20 with five extreme outliers, B3.22 and B3.24 with 3 extreme outliers, B3.23 with 6 extreme outliers, and B4.25 with 8 extreme outliers. The rest of the items had a number of mild outliers, ranging from one mild outlier to six mild outliers. These outliers were noted for further outlier assessment in multivariate level.

The items were checked for multivariate outlier using assessment normality and the value of skewness for all items was satisfied (−2 to 2) [13]. In addition, the value of multivariate kurtosis showed lower than 50.0 which supports the data normality [14]. The data were normally distributed; therefore, all 108 observations were retained and used for CFA analysis. A latent exogenous construct, that is, occupation which consists of four items, was eliminated in the full measurement model due to numerous missing values and a new conceptual framework of Malay version of IFSAC was created.

### 3.3. Construct Validity

**Figure 1:**
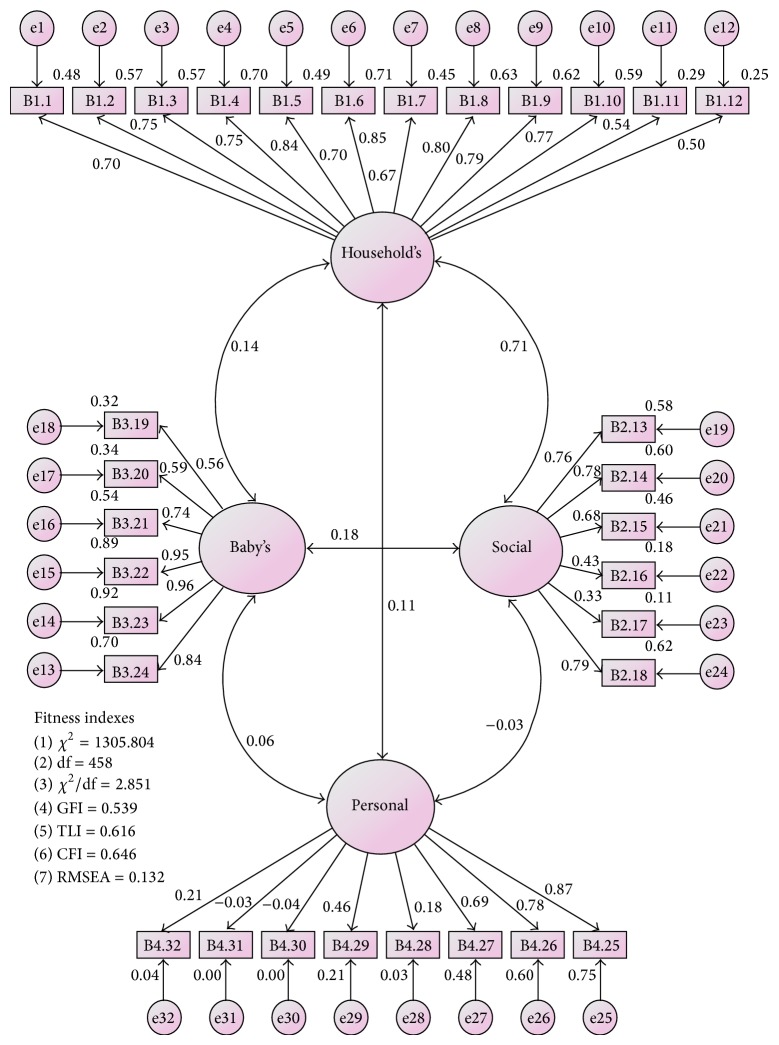
Malay version of IFSAC measurement model based on four factors structure (Model 1).

Five models were generated to achieve acceptable fitness. Model 1 (Figure 1) showed unacceptable fitness level (Table 3). Standardized factor loadings (standardized regression weights) for each item were identified after constructing the full measurement model to meet criteria fitness indexes. Nine items (B1.11, B1.12, B2.16, B2.17, B4.28, B4.29, B4.30, B4.31, and B4.32) with factor loading of <0.6 (ranging −0.04 to 0.53) were removed one by one, leaving 23 items for Model 2.

Model 2 showed unacceptable fitness level. Two items (B3.19 and B3.20) were decided to be retained in the measurement model because these items were acceptable based on terms of content; thus, modification indices (MI) was referred to examine the redundant items in the measurement model. The modification indices of less than 15 were acceptable for all items except for e1–e5 (18.558), e7-e8 (31.897), e7–e9 (19.043), e8-e9 (53.310), e7–e19 (20.669), and e17-e18 (82.014). There were two choices to address this problem. First, delete one of these two redundant items and respecify the measurement model. Second, set these two correlated errors to be “free parameter estimate” and respecify the measurement model [14]. Thus, e17 (B3.20)-e18 (B3.19) was set as free parameter estimates in Model 3.

Model 3 still showed unacceptable fitness level and three items were further deleted. Item e1 (B1.1) was deleted because of lower factor loading (0.73) compared to item e5 (B1.5) (0.74), while items e7 (B1.7) and e9 (B1.9) were removed from the model due to the presence of more than one covariance between each pair of items. This resulted in Model 4; however, fitness level was still not achieved (Table 3).

**Figure 2:**
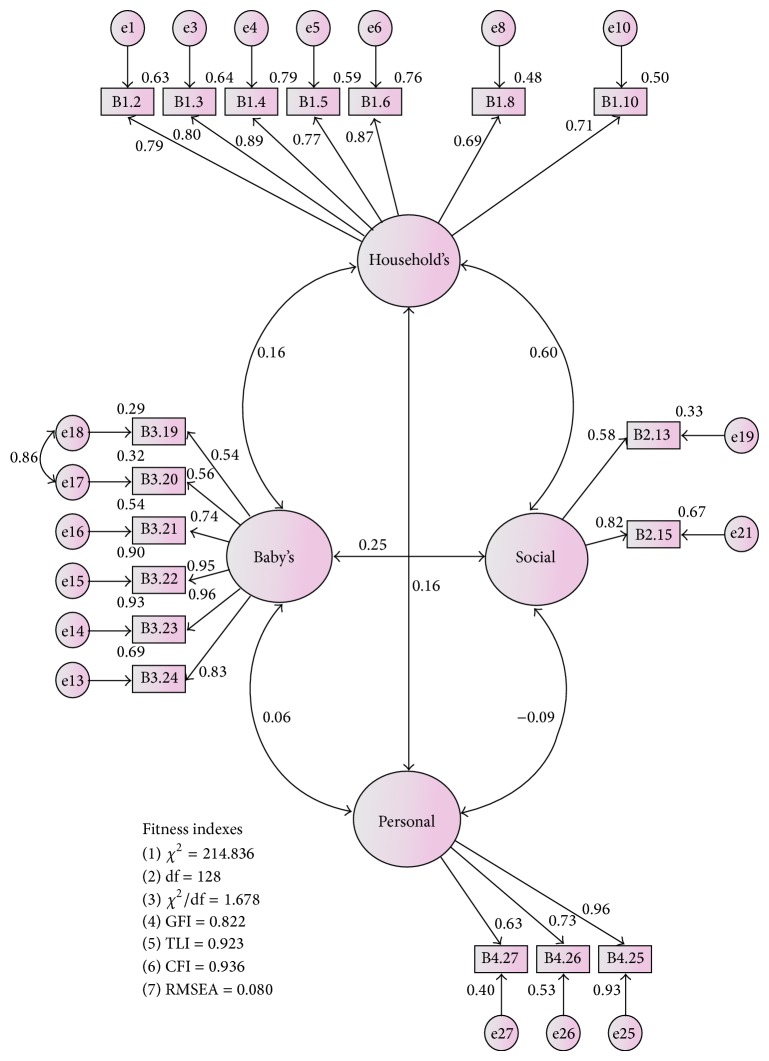
Malay version of IFSAC measurement model after removal of two items based on irrelevant content of item to factor (Model 5).

The present study found that two items had irrelevant content. The irrelevant content of two items were “B2.14: professional organization” and “B2.18: social clubs” with the value of 0.84 and 0.78, respectively. The content was not acceptable in Malaysian culture in which majority of mothers after childbirth did not get involved in any association or services during the confinement period. Hence, these items (B2.14 and B2.15) were deleted from the model to achieve the fitness level required (Table 3) in Model 5 (Figure 2). Model 5 was accepted as the final model with four constructs and 18 items because it demonstrated acceptable factor loadings, domain-to-domain correlation, and best fit (the Appendix).

### 3.4. Discriminant Validity

According to Zainudin (2012), discriminant validity is achieved when a diagonal value in bold is higher than the values in its row and column (Table 4). The diagonal value in bold is the square root of AVE while other values are the correlation between the constructs. Based on Table 5, the values of diagonal of constructs (in bold) were higher than other values of correlation between constructs (other values in its row and column). Thus, discriminant validity was achieved [14].

### 3.5. Convergent Validity

Convergent validity assessed the items related to the proposed construct. The AVE is a summary measure of convergent among the items. It is acceptable when value of AVE > 0.5 [14]. Table 5 showed AVE of household (0.628), baby (0.610), and personal care (0.617) constructs with adequate convergent. Social construct demonstrated an AVE of 0.499 that was considered as close to adequate convergent.

### 3.6. Reliability

Table 5 showed all four constructs (household, social, infant care, and personal care) had a measure of good reliability, as their composite reliability was 0.921, 0.659, 0.9, and 0.824, respectively. Three constructs (household, infant care, and personal care) with 0.92, 0.907, and 0.813, respectively, have Cronbach's alpha higher than 0.7. In addition, social construct also was considered as satisfactory reliability as its Cronbach's alpha values were close to 0.7 (0.626). Thus, reliability for this model was satisfied.

### 3.7. Concurrent Criterion Validity

Pearson's product moment correlation coefficient was *r* = 0.324 (*P* = 0.001). The criterion validity is demonstrated because the validated IFSAC correlates well with the criterion. The scores from IFSAC were directly related to the PCS scores of SF12 in assessing the physical function of the postpartum mothers.

## 4. Discussion

This study was successfully translated and validates the IFSAC into Malay language and it is applicable to be used for Malay postpartum women. Translation process was performed with the aim to achieve equivalence between the original English and translated Malay version. Item relevancy and acceptability, content, and semantic and conceptual equivalence were considered throughout the process. In terms of semantic meaning, the respondents were unable to differentiate the meaning in three items in the household care domain. Thus, local examples were added to each item in order to enhance the meaning of the items.

The original IFSAC consists of five domains to assess functional status of postpartum mother, namely, household activities, social and community activities, infant care, personal care, and occupational activities. However, based on this Malay version of IFSAC tested in the Malaysian culture, only four from five domains were being analyzed for CFA. All four items content in domain occupation were identified to be not relevant in this study setting because generally Malaysian mothers did not start working within a month or 40 days postpartum as they were still in the confinement period. Thus, in Malaysian setting, the household activities, social and community activities, infant care, and personal care are acceptability adequate to assess functional status of postpartum mothers.

In addition to that, the experts panel did identify few items in the social activities domains which portrayed some aspects that were not culturally acceptable but had agreed to retain all the items and to be further tested in the factor analysis. In fact, the respondents revealed that they had difficulty to appreciate the items in the social activities domains in their daily life. These items, namely, “B2.14: professional organization” and “B2.18: social clubs,” were decided to be deleted from the model due to its irrelevancy to local culture and the removal of the items advantageously leads to better fitness of the model. The items were not acceptable in Malaysian culture in which majority of mothers after childbirth did not get involved in any association or services during the confinement period.

The conceptual measurement was further tested using CFA validity procedure using AMOS. Confirmatory factor analysis based on maximum likelihood method (ML) is a parametric procedure and it relies on a number of assumptions which are basically similar to linear regression analysis [20]. The repeated processes of modification were performed based on the factor loadings, correlation between domains, and model fitting. Additionally, consideration of content relatedness was applied at each step of modification. Degree of redundancy and relevance of the items to the factor was used together with the statistical criteria as a means for item removal [20].

The initial model of 32 items was loaded into four domains and the final model with 18 items loaded into four domains and exhibited most acceptable fit (RMSEA = 0.08, GFI = 0.822, CFI = 0.936, TLI = 0.923, and *χ* ^2^/df = 1.678). In general, the confirmatory factor analysis suggested that the final model with 18 items had the best fit. All the CFA goodness-of-fit indices which include RMSEA, GFI, CFI, TLI, and *χ* ^2^/df supported the model fitness.

The factor loading initial model evaluation was followed by convergent and discriminant validity. The convergent validity assessed the items related to the proposed construct. The AVE is a summary measure of convergent among the items. All the domains in the present study showed an AVE of 0.5 and above indicating adequate convergent. Therefore, all domains indicate that the items were well correlated with the construct.

CFA in present study also tested the discriminant validity of constructs through the intercorrelations among the latent factors [21]. The discriminant validity is referred to the construct and should not correlate with dissimilar or unrelated variables [22]. It was presented as the square root of AVE and these values were higher than the correlation values between the constructs.

Final evaluation involves reliability analysis. Reliability refers to the accuracy and precision of a measurement procedure [23]. The present study demonstrated that all constructs had good constructs reliability as the composite reliability for household activities, social and community activities, infant care, and personal care were 0.921, 0.659, 0.900, and 0.824, respectively. Cronbach's alpha for household, infant care, and personal care domains was highly satisfactory except for social and community domain that was slightly low (Cronbach's alpha 0.626). In general, composite reliability based on CFA and Cronbach's alpha support the internal consistency of the scales.

## 5. Limitation of the Study

Several limitations were encountered. Firstly, cultural differences showed that the original construct was not able to be maintained in our population. In addition, original author did not perform CFA and, therefore, our results differ from the original study.

## 6. Conclusions

The Malay version IFSAC has achieved the content validity through translational process and expert review. The confirmatory factor analysis showed that the final model with 18 items had a good fit (RMSEA = 0.08, GFI = 0.822, CFI = 0.936, TLI = 0.923, and *χ* ^2^/df = 1.678). The four constructs had showed a measure of good convergent validity, discriminant validity, internal reliability, and construct reliability. Replication in other study population is recommended to confirm the structure and testing its invariance across samples. This would provide further evidence to support the arrangement of the Malay version IFSAC items.

### 6.1. Clinical Implications

Consider the following:

- validation of the IFSAC questionnaire performed for the first time in Malaysia;
- forward and back translation of the IFSAC questionnaire to Bahasa Melayu;
- validity and reliability of the Bahasa Melayu version of the IFSAC questionnaire;
- validation using confirmatory factor analysis based on structural equation modelling;
- IFSAC measures functional status during recovery from childbirth;
- assist assessment of functional status after childbirth for health personnel especially midwives.

## Figures and Tables

**Table 1:** IFSAC subscales and items.

Item	Subscale
	(1) *Household activities *
B1.1	Care of family members
B1.2	Cleaning the house
B1.3	Tidying the house (making beds, picking up things, etc.)
B1.4	Laundry
B1.5	Doing dishes
B1.6	Cooking
B1.7	Household business (paying bills, banking, etc.)
B1.8	Grocery shopping
B1.9	Shopping, other than groceries
B1.10	Doing errands
B1.11	Heavy housework and maintenance work (seasonal cleaning, painting, etc.)
B1.12	Caring for pets
	(2)* Social and community activities *
B2.13	Community service organizations
B2.14	Professional organization
B2.15	Religious organizations
B2.16	Socializing with friends
B2.17	Socializing with relatives
B2.18	Social clubs
	(3)* Infant care *
B3.19	Daytime feedings
B3.20	Night feedings
B3.21	Bathe the baby
B3.22	Change diapers
B3.23	Change the baby's clothes
B3.24	Play with the baby
	(4)* Personal care *
B4.25	Spend much of the day lying down
B4.26	Sit during much of the day
B4.27	Spend much of the day sleeping or dozing
B4.28	Stand for only short periods of time
B4.29	Spend most of the day in my nightgown/bathrobe
B4.30	Take walks
B4.31	Go up and downs stairs
B4.32	Walk slowly
	(5)* Occupational activities *
B5.33	Am accomplishing as much as usual in my job
B5.34	Act irritable toward my work associates (give sharp answers…)
B5.35	Am working shorter hours
B5.36	Am doing my job as carefully and accurately as usual

**Table 2:** Sociodemographic characteristics.

Sociodemographic characteristics	Mean (SD^†^)	*n* (%)
Age (year)	31.4 (6.02)	
Household income (RM/month)	3000 (1750)^‡^	
Duration of marriage (year)	5 (8)^‡^	
Parity	2 (3)^‡^	
Number of children	2 (2)^‡^	
Occupation		
Working		66 (61.1)
Not working		42 (38.9)

^†^Standard deviation.

^‡^Median (interquartile range) skewed to the right.

**Table 3:** Fitness level of Models 1–5.

Model	RMSEA	GFI	CFI	TLI	*χ* ^2^/df
Model 1	0.132	0.539	0.646	0.616	**2.851**
Model 2	0.144	0.638	0.746	0.713	**3.204**
Model 3	0.121	0.669	0.82	0.769	**2.567**
Model 4	0.092	0.782	0.906	0.890	**1.897**
Model 5	**0.080**	0.822	**0.936**	**0.923**	**1.678**

RMSEA: root mean square error of approximation.

GFI: goodness-of-fit index.

CFI: comparative fit index.

TLI: Tucker-Lewis index.

*χ* ^2^/df: Chi-squared/degree of freedom.

**Table 4:** The CFA results for discriminant validity.

Construct	Household's	Social	Infant care	Personal care
Household	**0.79**			
Social	0.60	**0.71**		
Infant care	0.16	0.25	**0.78**	
Personal care	0.16	−0.09	0.06	**0.79**

**Table 5:** The CFA validity and reliability results for final model (Model 5).

Construct	Item	Factor loading	Cronbach's alpha (≥0.7)	CR (≥0.6)	AVE (≥0.5)
Household	B1.2	0.79	0.920	0.921	**0.628**
	B1.3	0.8			
	B1.4	0.89			
	B1.5	0.77			
	B1.6	0.87			
	B1.8	0.69			
	B1.10	0.71			

Social	B2.13	0.58	0.626	0.659	**0.499**
	B2.15	0.82			

Infant care	B3.19	0.54	0.907	0.900	**0.61**
	B3.20	0.56			
	B3.21	0.74			
	B3.22	0.95			
	B3.23	0.96			
	B3.24	0.83			

Personal care	B4.25	0.96	0.813	0.824	**0.617**
	B4.26	0.73			
	B4.27	0.63			

AVE: average variance extracted.

CR: composite reliability.

**Table 6:** Please check all the usual *household responsibilities* you had *prior* to the baby's birth and then indicate to what extent you have resumed these responsibilities *since* the baby was born.

Number	Perkara	**Belum **lakukan seperti sebelum bersalin	**Mula** lakukan seperti sebelum bersalin	Lakukan **sebahagian** seperti sebelum bersalin	Lakukan **sepenuhnya** seperti sebelum bersalin
(1)	Membersihkan rumah (cth: menyapu, mengemop, dll)	1	2	3	4
(2)	Mengemas rumah (cth: mengemas katil, mengutip barang-barang yang berselerak, dll)	1	2	3	4
(3)	Membasuh baju	1	2	3	4
(4)	Menyediakan minuman	1	2	3	4
(5)	Memasak	1	2	3	4
(6)	Membeli barangan runcit	1	2	3	4
(7)	Melakukan apa-apa kerja yang disuruh	1	2	3	4

Comments:

**Table 7:** Please check all the usual *social and community activities* you did *prior* to the baby's birth and then indicate to what extent you have resumed these responsibilities *since* the baby was born.

Number	Perkara	**Belum** lakukan seperti sebelum bersalin	**Mula** lakukan seperti sebelum bersalin	Lakukan **sebahagian** seperti sebelum bersalin	Lakukan **sepenuhnya** seperti sebelum bersalin
(8)	Khidmat masyarakat (cth: gotong-royong)	1	2	3	4
(9)	Aktiviti keagamaan (cth: berjemaah/mendengar ceramah di masjid)	1	2	3	4

Comments:

**Table 8:** Please *circle the number* that indicates to what extent you have assumed your part of the following aspects of the *baby's care*.

Number	Perkara	Tidak pernah	Kadang-kadang	Kebanyakan masa	Setiap kali
(10)	Menyusu bayi di siang hari	1	2	3	4
(11)	Menyusu bayi di malam hari	1	2	3	4
(12)	Memandikan bayi	1	2	3	4
(13)	Menukar lampin bayi	1	2	3	4
(14)	Menukar pakaian bayi	1	2	3	4
(15)	Bermain bersama bayi	1	2	3	4

Comments:

**Table 9:** Please respond to the following phrases based on how your life has been during the *past week or two*.

Number	Perkara	Tidak pernah	Kadang-kadang	Kebanyakan masa	Setiap masa
(16)	Berbaring sepanjang hari	4	3	2	1
(17)	Duduk sepanjang hari	4	3	2	1
(18)	Tidur sepanjang hari	4	3	2	1

Comments:

## Appendix

Malay Version of the Inventory of Functional Status after Childbirth Questionnaire.

Please think about the time since the birth of your baby and then respond to the items shown in Tables 6, 7, 8, and 9.

## Abbreviations

AMOS: Analysis of moment structure
AVE: Average variance extracted
CFA: Confirmatory factor analysis
CFI: Comparative fit index
CR: Composite reliability
GFI: Goodness of fit index
IFSAC: Inventory of Functional Status after Childbirth
MI: Modification indices
ML: Maximum likelihood method
PCS: Physical component summary
RMSEA: Root mean square error of approximation
SD: Standard deviation
SF-12: 12-item Short Form Health Survey
SF-36: 36-item Short Form Health Survey
SEM: Structural equation modelling
SPSS: Statistical packages for the social sciences
TLI: Tucker-Lewis index
*χ*^2^/df: Chi-squared/degree of freedom.
